# 16S rRNA gene sequencing of microbiota from the preen oil and cloaca of chipping sparrows (*Spizella passerina*)

**DOI:** 10.1128/mra.00576-24

**Published:** 2024-08-30

**Authors:** Tricia A. Van Laar, Jonathan M. Greenberg, Kevin R. Theis, Danielle J. Whittaker, Joel W. G. Slade

**Affiliations:** 1 Department of Biological Sciences, California State University, Stanislaus, Turlock, California, USA; 2 Department of Psychiatry & Behavioral Neurosciences, Wayne State University School of Medicine, Detroit, Michigan, USA; 3 Department of Research and Development Service, John D. Dingell Veterans Affairs (VA) Medical Center, Detroit, Michigan, USA; 4 Department of Biochemistry, Microbiology, and Immunology, Wayne State University, Detroit, Michigan, USA; 5 College of Earth, Ocean, and Atmospheric Sciences, Oregon State University, Corvallis, Oregon, USA; 6 Department of Biology, California State University, Fresno, California, USA; California State University, San Marcos, California, USA

**Keywords:** microbiome, preen oil, cloaca, chipping sparrow, *Spizella passerina*

## Abstract

We present the results of 16S rRNA gene amplicon sequencing of the microbiota from preen oil and the cloaca of chipping sparrows (*Spizella passerina*) collected near Mountain Lake Biological Station in Pembroke, VA.

## ANNOUNCEMENT

New world sparrows (Passerellidae), specifically non-migratory dark-eyed juncos (*Junco hyemalis carolinensis*), are at the forefront of avian microbial ecology studies (1). These birds harbor symbiotic bacteria used for chemical communication through preen oil (2). In the same habitat is one overlooked species, the migratory chipping sparrow (*Spizella passerina*). Here, we describe microbial communities of the preen oil and cloaca of chipping sparrows to provide information for future interspecific comparative studies.

Birds were sampled as previously described (2). Microbial communities from the preen oil and cloaca were collected using a pre-moistened swab with sterile buffer (20 mM Tris pH 8, 2 mM EDTA, and 1.2% Triton X-100). We extracted DNA with the Qiagen DNeasy Powerlyzer PowerSoil DNA Isolation Kit with the following modifications: (i) Swabs were soaked in a 500-µL bead solution and 200-µL phenol:chloroform:isoamyl alcohol for 10 min before using Biospec Products MiniBeadBeater-16 run 2× for 30 sec. (ii) Samples received 100 µL each of solutions C2 and C3, plus 1-µL RNase A, and incubated at 4°C for 5 min before one-step centrifugation. (iii) Lysates were mixed with 650-µL solution C4 and 650-µL 100% ethanol instead of using 1,200-µL solution C4 alone. (iv) DNA was eluted in 60-µL solution C6, reduced from 100 µL (1). We amplified bacterial DNA using nested PCR as described previously (2). The amplified V4 region of the 16S rRNA gene was prepared using the V2 500 cycle MiSeq Reagent Kit (Illumina MS102-2003) and sequenced on the Illumina MiSeq platform by Michigan State University Research Technology Support Facility’s Genomics Core generating 2 × 250-bp reads.

Analyses were performed using R Statistical Software v4.3.3 (3). We used DADA2 v1.30.0 (4) to process sequencing reads. Default parameters for DADA2 were used except reads were trimmed 10 bp at the 5′ end and truncated at 240 bp (F) and 200 bp (R) at the 3′ end. Paired-end reads were merged, and chimeric sequences were removed. Table 1 tracks reads through the DADA2 pipeline. We assigned taxonomy using the SILVA 138.1 data set with species information (5). Contaminating sequences from blank and water extractions were removed using decontam v1.22.0 (6). We used phyloseq v1.46.0 (7) to analyze alpha (observed amplicon sequence variants, Shannon diversity, and Simpson’s diversity index) and beta (Bray–Curtis dissimilarity) diversity. We used vegan v2.6.6.1 (8) for statistical analyses and ggplot2 v3.5.1 (9) to generate figures.

**TABLE 1:** Sample information for sequencing reads

Bird	Sample	Site	Input	Filtered	Denoised F	Denoised R	Merged	Non-chimera	NCBI accession
CHSP02	262	Cloaca	23,692	21,166	20,744	20,850	19,894	18,729	SRR29202452
CHSP03	8	Cloaca	53,394	48,310	46,990	47,125	43,746	42,312	SRR29202442
CHSP04	39	Cloaca	11,372	9,649	9,344	9,322	8,763	8,707	SRR29202444
CHSP05	2	Cloaca	40,953	35,685	34,840	34,926	33,020	31,999	SRR29202438
CHSP06	377	Cloaca	58,412	53,359	52,344	52,381	50,307	49,818	SRR29202445
CHSP07	372	Cloaca	45,801	42,088	41,404	41,524	40,076	39,548	SRR29202446
CHSP08	20	Cloaca	54,567	45,840	44,686	44,668	40,451	39,019	SRR29202437
CHSP09	184	Cloaca	55,020	48,926	48,163	48,153	46,358	44,916	SRR29202440
CHSP10	214	Cloaca	19,470	18,049	17,818	17,853	17,483	17,483	SRR29202435
CHSP11	180	Cloaca	112,134	100,851	100,044	100,117	89,998	88,560	SRR29202443
CHSP12	186	Cloaca	29,577	25,491	25,041	25,060	23,672	22,923	SRR29202439
CHSP02	123	Preen	3,984	3,585	3,452	3,488	3,244	3,202	SRR29202454
CHSP03	298	Preen	9,871	9,269	9,186	9,208	9,144	6,304	SRR29202450
CHSP04	93	Preen	11,302	10,028	9,851	9,844	9,332	9,012	SRR29202441
CHSP05	103	Preen	43,007	38,430	37,808	37,882	36,601	35,407	SRR29202455
CHSP06	237	Preen	38,532	34,316	33,689	33,706	32,041	30,803	SRR29202453
CHSP07	207	Preen	8,057	7,037	6,860	6,878	6,591	6,542	SRR29202436
CHSP08	283	Preen	1,655	1,461	1,379	1,374	1,308	1,308	SRR29202451
CHSP09	319	Preen	20,659	18,642	18,256	18,305	17,466	17,133	SRR29202449
CHSP10	326	Preen	44,570	40,618	39,818	39,740	37,751	37,589	SRR29202448
CHSP11	22	Preen	59,017	53,469	52,934	52,982	48,990	48,558	SRR29202434
CHSP12	329	Preen	40,065	36,088	35,652	35,645	34,274	33,466	SRR29202447

**Fig 1:**
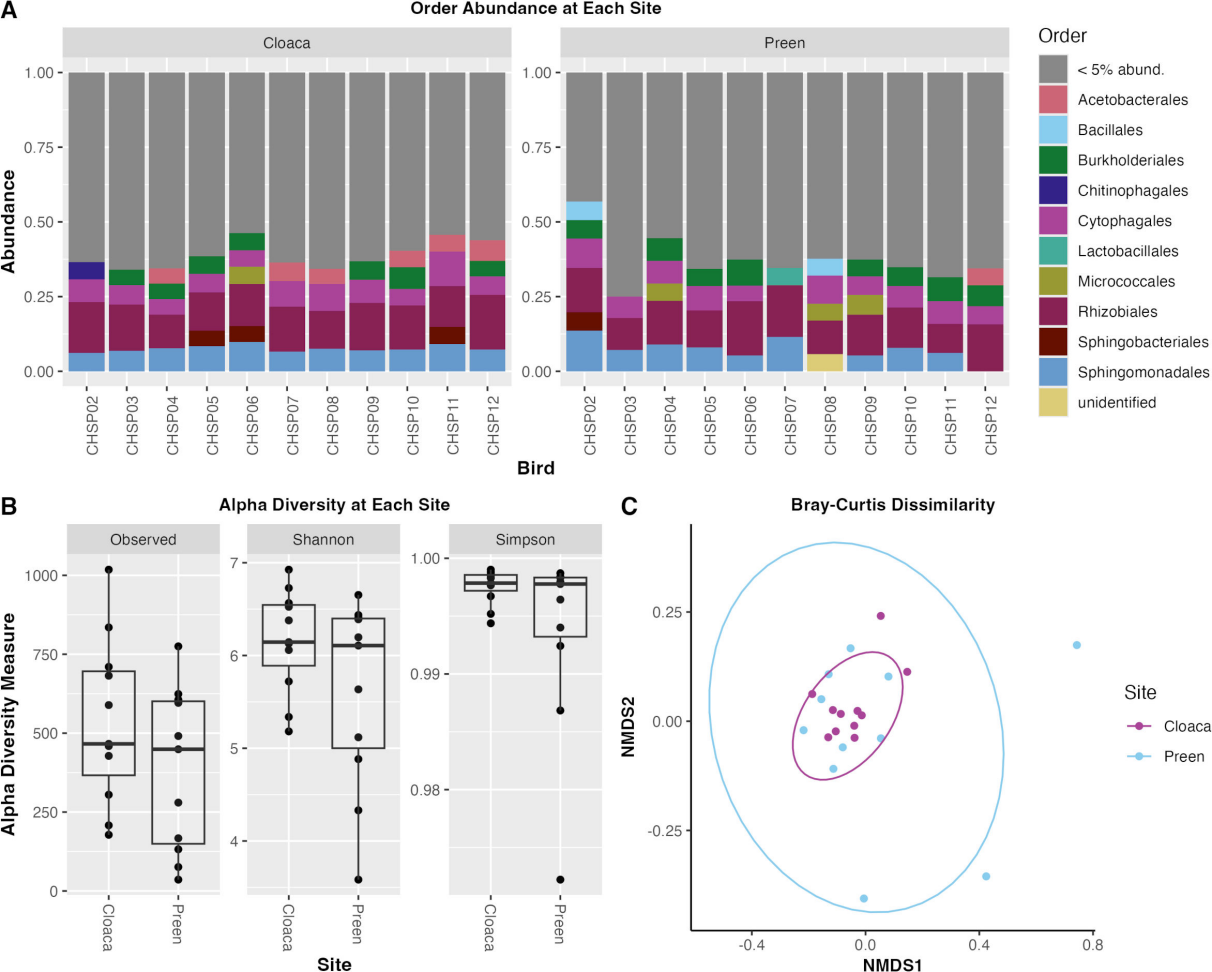
Microbial diversity and community composition in cloaca and preen gland samples from chipping sparrows. (**A**) Relative abundance of orders obtained from 16S rRNA gene sequencing of preen oil and the cloaca. Orders with less than 5% abundance were grouped together as were orders that were unidentified. (**B**) Alpha diversity of cloaca and preen oil communities. (**C**) Non-metric multidimensional scaling (NMDS) plot of Bray–Curtis dissimilarity.

A column chart comparing relative order abundance between preen oil and cloaca showed no noticeable differences (Fig. 1A). The Similarity Percentages function (simper) did not identify any statistically significantly different taxa in preen oil compared to cloaca. Alpha diversity analysis showed that the preen oil community was less diverse than that of the cloaca, though not significantly (Fig. 1B). We saw no significant difference in Bray–Curtis dissimilarity between the preen oil and cloaca communities (Fig. 1C).

## Data Availability

The 16S rRNA gene amplicon sequences have been deposited in the GenBank Sequence Read Archive (SRA) under the BioProject accession number PRJNA1117373 under the SRA accession numbers SRR29202434- SRR29202455.
